# Odontological Society of New York

**Published:** 1884-04

**Authors:** 


### ODONTOLOGICAL SOCIETY OF NEW YORK.

The New York Odontological Society met Tuesday evening, February 19th, at the residence of Dr. W. E. Hoag.

*Dr. N. W. Kingsley*—Exhibited artificial teeth mounted on a metallic base, composed of sixteen parts of pure tin with one part of bismuth. This compound flows at a temperature of less than four hundred degrees, and in a moulten state is poured into a matrix composed of sea-sand and plaster, in which the teeth as arranged are imbedded. The specimens were exceedingly beautiful, and show a decided advance in the production of artificial dentures. Dr. K. stated that they could be easily repaired, and where plain teeth were required the gum could be formed of pink vulcanite. He also asserted that the material would retain its color and brilliancy better even than gold.

*Dr. J. Morgan Howe*—Read a paper on Iodoform, wherein he referred to his success in the use of this remedial agent in cases of alveolar abscess. He recommends a solution of iodoform in equal parts of sulphuric ether and alcohol.

The essayist for the evening was *Prof. Thos. Fillebrown*, of Boston, who read a paper on the advantages of gold foil as a filling material; he also gave his particular method of manipulating it. He recommends number four cohesive foil, which he cuts into strips of various widths and converts into coils. These he warms over a spirit lamp, avoiding red heat, and carries each required coil to the cavity, carefully packing and lapping fold after fold with a gentle pressure, which might be described as semi-rotary, at the same time rubbing the foil against the cavity walls.

This method, he claims, will bring the layers of gold together so admirably that perfect cohesion is secured. Prof. F. exhibited instruments which he employs in carrying out his method of manipulating or rubbing the coils of foil into tooth cavities, the "points," or packing surfaces of which, were slightly rounded to get rid of sharp edges, and with exceedingly fine serrations to prevent slipping.*

* This principle of packing is somewhat similar to that suggested some years ago by Dr. Shumway, of Massachusetts, and later by Dr. Chance, of Oregon; the former, however, condensing with ivory, and the latter with gold points.

Prof. F. dilated largely upon the nice cohesive properties of gold foil, its soft plastic nature, etc., but emphatically condemned the practice of punching, wedging or malleting gold into a cavity, which, he says, beats out and destroys the best properties it possesses. He considers gold foil preferable to all other stoppings, both as to appearance and durability.

*Dr. A. H. Brockway*—Stated that he believed many cases occurred where plastic stoppings answered a most excellent purpose. He also spoke of the success he had experienced in the use of "prepared" or "sponge" gold, and wondered why it was not in more common use by dentists.

*Dr. J. B. Rich*—Favored the use of cohesive foil, preferring number four, but could see no sense in rolling it into coils. He claimed that, in order to pack it perfectly, it should be folded into ribbons, thus permitting the flattened layers to cohere compactly together, making good solid fillings.

*Dr. E. A. Bogue*—Mildly intimated that, although gold fillings might be desirable, cases were very common where individuals, anxious to save their teeth, were unable to indulge in such expensive luxuries as gold fillings, and that their needs should be considered. Dr. B. contended that much excellent work had been done with non-cohesive foil, and hand-pressure instruments. Many old-fashioned cylinder fillings had done, and were still doing, good service. He also instanced an interview with a manufacturer of gold foil, who asserted that varying degrees of cohesion could be imparted to portions cut from a single bar of gold, by remelting each portion with different substances used as flux.

